# *Bacillus thuringiensis* Cry1A toxins are versatile proteins with multiple modes of action: two distinct pre-pores are involved in toxicity

**DOI:** 10.1042/BJ20131408

**Published:** 2014-03-28

**Authors:** Isabel Gómez, Jorge Sánchez, Carlos Muñoz-Garay, Violeta Matus, Sarjeet S. Gill, Mario Soberón, Alejandra Bravo

**Affiliations:** *Instituto de Biotecnología, Universidad Nacional Autónoma de México, Apdo. Postal 510-3, Cuernavaca 62250, Morelos, Mexico; †Department of Cell Biology and Neuroscience, University of California, Riverside, Riverside, CA 92521, U.S.A.

**Keywords:** *Bacillus thuringiensis*, Cry toxin, mechanism of action, oligomerization, pore-forming toxin, pre-pore, ABCC2, ATP-binding cassette C2, ALP, alkaline phosphatase, APN, aminopeptidase N, BBMV, brush border membrane vesicle, 3d-Cry, three-domain Cry, PC, phosphatidylcholine, PE, phosphatidylethanolamine, PFT, pore-forming toxin, PLB, planar lipid bilayer, SUV, small unilamellar vesicle

## Abstract

Cry proteins from *Bacillus thuringiensis* are insecticidal PFTs (pore-forming toxins). In the present study, we show that two distinct functional pre-pores of Cry1Ab are formed after binding of the protoxin or the protease-activated toxin to the cadherin receptor, but before membrane insertion. Both pre-pores actively induce pore formation, although with different characteristics, and contribute to the insecticidal activity. We also analysed the oligomerization of the mutant Cry1AbMod protein. This mutant kills different insect populations that are resistant to Cry toxins, but lost potency against susceptible insects. We found that the Cry1AbMod-protoxin efficiently induces oligomerization, but not the activated Cry1AbMod-toxin, explaining the loss of potency of Cry1AbMod against susceptible insects. These data are relevant for the future control of insects resistant to Cry proteins. Our data support the pore-formation model involving sequential interaction with different midgut proteins, leading to pore formation in the target membrane. We propose that not only different insect targets could have different receptors, but also different midgut proteases that would influence the rate of protoxin/toxin activation. It is possible that the two pre-pore structures could have been selected for in evolution, since they have differential roles in toxicity against selected targets, increasing their range of action. These data assign a functional role for the protoxin fragment of Cry PFTs that was not understood previously. Most PFTs produced by other bacteria are secreted as protoxins that require activation before oligomerization, to finally form a pore. Thus different pre-pores could be also part of the general mechanism of action of other PFTs.

## Abstract 2

Two distinct functional pre-pore oligomers of the Cry1Ab insecticidal toxin are formed before membrane insertion. These oligomers are formed after binding of either the protoxin or the protease-activated toxin to the cadherin receptor. Both pre-pores have different characteristics and contribute to insecticidal activity.

## INTRODUCTION

PFTs (pore-forming toxins) are one of the most important groups of virulent factors that are critical for the effective pathogen attack of their targets [1,2]. Different pathogenic bacteria and mushrooms produce them. In addition, the mammalian perforin, or C9 from the complement system, generates pores in target pathogens [3]. PFTs are also produced by *Bacillus thuringiensis*, during the sporulation phase of growth. These bacteria produce Cry and Cyt PFTs that are active against different insect pests in agriculture or are vectors of human diseases [4]. Of the many groups of Cry toxins presently described (see http://www.btnomenclature.info/), the 3d-Cry (three-domain Cry) toxins represent the largest family. Several members of these 3d-Cry toxins have been expressed in transgenic plants, resulting not only in efficient control of insect pests, but also in significant reduction in the use of chemical insecticides in the field [6,7].

The loss of PFT activity results in significant reduction of virulence in diverse pathogens [1,2]. Thus understanding the mechanism of membrane insertion of these proteins would address a fundamental question in biology. Different models have been proposed to describe how the pores are formed in the target membranes before causing osmotic lysis and cell death [1–4]. The main unsolved questions are focused on how PFTs affect the target membranes, in particular whether sequential interactions with different receptors are required *in vivo*, and whether the pore-forming oligomer is assembled before or after the monomeric toxin is inserted into the membrane.

In the case of Cry1A PFT, one model proposes a sequential binding interaction of the toxin with different midgut proteins (receptors), leading to the assembly of a pre-pore oligomeric structure before it inserts into the membrane and forms a pore. The first step in this model is a low-affinity interaction of monomeric activated toxin with APN (aminopeptidase N) or ALP (alkaline phosphatase) receptors (*K*_d_ 100–200 nM), which are highly abundant in the membrane [8,9]. This important step locates the toxin in close proximity to the microvilli membrane. The second step is a high-affinity interaction with a cadherin receptor (*K*_d_ 1 nM) that involves interaction of three different epitopes, both in the toxin and in the cadherin molecule [10,11]. This complex interaction facilitates an additional cleavage at the N-terminal end of the toxin, triggering oligomerization of the protein [12,13]. The oligomeric structure gains a 100–200-fold higher binding affinity to APN and ALP receptors (*K*_d_ 0.5 nM) [8,9], facilitating the insertion of the pre-pore into membrane lipid rafts [12,13] forming the lytic pore in the midgut cells [8,12].

The requirement for cleavage of the N-terminal region to form oligomeric structures responsible for pore-formation activity was questioned [14] because Cry1A, Cry1C and Cry4Ba monomers are able to form pores *in vitro*, in PLBs (planar lipid bilayers), in the absence of receptors or proteases [15–24]. Hence an alternative model has been proposed where the pore is formed after insertion of activated monomeric toxin into the membrane [14]. In this model, interaction with any one of the toxin receptors that are present in insect midgut cells, such as cadherin, APN or ALP, would facilitate toxin insertion into the membrane. After monomeric toxin inserts into the membrane, oligomerization follows in the membrane plane, forming pores that kill cells [14].

However, there are significant problems with the latter model. For example, insertion of trypsin-activated toxin into the bilayer was not efficient, since, in some cases, pores failed to appear at all and/or required special activation processes as well as special procedures to incorporate the toxin into the bilayer such as disruption and repainting the bilayer or microinjection of the toxin directly into the hole of the bilayer chamber followed by chloroform evaporation [15,19,21,23,24]. In addition, the pores that were formed displayed several subconducting states [16–21,24] and a variety of kinetic behaviours, such as complex activity patterns, rapid flickering or slow gating and long-lasting subconducting states, were observed [19–21]. Furthermore, most of the reported records of the pores induced by these toxins last only for 1–4 s [14,15,17–24], showing bursts of up to ten different levels [18,20] as well as toxin washout of the membrane [16].

In contrast, in the sequential bind model, it is proposed that a pre-pore is formed after activation of Cry1Ab protoxin in the presence of cadherin receptor forming a 250 kDa structure. The size-exclusion-purified Cry1Ab 250 kDa oligomers readily incorporate into the bilayer, inducing ion pores with a stable conductance state and high open probability [16,25–27] for several minutes [16]. This pore activity could be observed at 20-fold lower toxin concentrations (6 nM) than that observed with monomeric trypsin-activated toxin under the same conditions (150 nM), indicating that insertion of pure oligomeric structures is more efficient than that of the monomeric toxin [16]. Nevertheless, direct comparison of pore formation between trypsin-activated toxin and oligomeric structures of Cry toxin has only been presented in one previous report [16] and more studies are needed.

In the present study, we provide further evidence in support of the sequential binding model. We show that Cry1Ab protoxin or activated toxin bind cadherin with similar affinities and two different pre-pores are produced depending on which of these molecules interacts with cadherin in the presence of insect midgut proteases. These pre-pores differ in their apparent size, sensitivity to temperature, ability to insert into synthetic membranes and pore characteristics. Our data suggest that the mechanism of action of Cry PFTs is complex, showing multiple mechanisms of action.

## EXPERIMENTAL

### Cry1Ab toxin purification

*B. thuringiensis* strains expressing Cry1Ab, Cry1Ab F371A [8,28] or Cry1AbMod [29] were grown for 3 days at 30°C in nutrient broth sporulation medium, supplemented with erythromycin at 10 μg·ml^−1^. After complete sporulation, the harvested products were washed twice in 300 mM NaCl and 10 mM EDTA, and crystal inclusions were purified using discontinuous sucrose gradients [30]. Protoxins were produced by solubilizing crystal inclusions in an alkaline buffer (50 mM Na_2_CO_3_ and 0.2% 2-mercaptoethanol, pH 10.5) for 2 h at 37°C. The soluble protoxins were recovered after centrifugation at ~18400 ***g*** for 20 min). Trypsin-activated toxins were obtained by treatment of soluble protoxin with trypsin [TPCK (tosylphenylalanylchloromethane)-treated trypsin from bovine pancreas (Sigma–Aldrich)] at a mass ratio of 1:50 (trypsin/toxin) for 2 h at 37°C after lowering the pH to 8.5 by adding 1 M Tris buffer (pH 8.5) at a 1:4 (v/v) ratio. PMSF (1 mM final concentration) was added to stop proteolysis. Activated proteins were purified by anion-exchange chromatography Mono Q–Sepharose fast flow (GE Healthcare) in an ÄKTA FPLC System (GE Healthcare), using a 50 mM Tris/HCl and 50 mM NaCl (pH 8.5) buffer, and a linear NaCl concentration gradient from 50 to 300 mM. Protein concentrations were determined by the method of Bradford, using BSA as a standard.

### Expression and purification of a cadherin fragment, ALP and APN

The *Manduca sexta* cadherin protein fragment (CR7–CR12) containing residues 810–1480 was expressed in *Escherichia coli* ER2566 cells as reported previously [26]. The cadherin fragment was purified using nickel affinity according to the manufacturer's instructions (Qiagen). APN and ALP proteins were produced in *E. coli* and purified as reported previously [31].

### ELISA binding assays

A 1 μg amount of the cadherin fragment suspended in 100 μl of PBS was used to coat each well of a 96-well ELISA plate, overnight at 4°C. Unbound cadherin fragments were removed and the ELISA plate was washed three times with 200 μl of PBS. Then, 200 μl of PBS, containing 2% (w/v) non-fat dried skimmed milk powder, was added and incubated at 37°C for 2 h. Each well was washed three times with PBS, followed by incubation with different toxin or protoxin dilutions in PBS for 1 h at 37°C. Excess unbound toxin was removed by two washing steps and each well was incubated with anti-Cry1Ab antibody (1/20000 dilution) for 1 h at 37°C. After washing, horseradish-peroxidase-conjugated rabbit antibody (1/10000 dilution) was added (37°C for 1 h). After additional washing, 100 μl of freshly prepared substrate solution composed of 5 mg of *o*-phenylenediamine (Sigma–Aldrich) in substrate buffer (0.1 M sodium phosphate, pH 5) plus 12 μl of 30% H_2_O_2_ (Sigma–Aldrich) was added and absorbance at 490 nm was measured. Data were analysed using Scatchard analysis with SigmaPlot (Systat Software).

### Analysis of oligomerization in solution

Oligomeric Cry1Ab structures were obtained by incubating 0.5 μg of the pure trypsin-activated toxin or 1.5 μg of protoxin with the cadherin fragment CR7–CR12 at a mass ratio of 1:4 (Cry1Ab/cadherin fragment) for 30 min at 37°C in a total volume of 100 μl of alkaline buffer. We used a higher amount of protoxin since half of it is degraded during activation with proteases. Subsequently the same mixture was incubated for 30 min in the presence of 0.5 μg of trypsin or 0.5 μl of midgut juice isolated from *M. sexta* as described in [32]. The reaction was stopped by adding 1 mM PMSF. Control samples that contained only the activated toxin or the protoxin, without cadherin or proteases, were included. Then, 4× Laemmli sample buffer (0.125 M Tris/HCl, 4% SDS, 20% glycerol, 10% 2-mercaptoethanol and 0.01% Bromophenol Blue) was added to the samples and they were divided into three tubes, each incubated for 5 min at a different temperature (25, 50 or 100°C). These samples were then separated by SDS/PAGE (8% gels) and electrotransferred on to PVDF membranes (Millipore), which were used for Western blot assays as described below.

### Incorporation of oligomers into SUVs (small unilamellar vesicles)

SUV liposomes were prepared as described previously [33] using PE (phosphatidylethanolamine), egg-yolk PC (phosphatidylcholine) and cholesterol (Avanti Polar Lipids) in a 7:3:2 molar proportion as described previously [14]. We selected this lipid composition since it was used previously to analyse pore-formation activity of different Cry toxins [17–19,20–24]. Lipids were hydrated in 10 mM Ches and 150 mM KCl (pH 9) at 2.6 μmol in 2.6 ml (1 mM final concentration) (1.52 μmol of PE; 0.65 μmol of PC and 0.43 μmol of cholesterol). SUVs were prepared by sonication in a Branson-1200 bath sonicator (Branson Ultrasonic) and used within 2 days of their preparation. Protoxin or activated toxin were incubated with cadherin fragment in the absence or presence of trypsin in solution as described above and these samples were incubated for 1 h at room temperature (25°C) with 50 μl of SUVs before analysing their pore formation. Samples were centrifuged at 157000  ***g*** for 30 min at 4°C, the supernatants were removed, and the pellets were washed with 100 μl of 10 mM Ches and 150 mM KCl (pH 9) and then centrifuged again. The final pellets were suspended in 60 μl of the same buffer and analysed by Western blotting as described below or suspended in 100 μl for pore-formation assays in black lipid bilayers as described below.

### *M. sexta* midgut BBMV (brush border membrane vesicle) purification

*M. sexta* midgut tissues from third instar larvae were dissected and stored immediately at −70°C. BBMVs were prepared by the magnesium precipitation method without protease inhibitors [34] and stored at −70°C until used. The BBMV protein concentrations were determined with the Lowry DC protein assay (Bio-Rad Laboratories) using BSA as a standard.

### Analysis of oligomerization in BBMVs

Cry1Ab oligomerization in BBMVs was analysed after incubation of 0.5 μg of Cry1Ab toxin or protoxin with 10 μg of BBMV protein for 1 h at 37°C in a total volume of 50 μl of alkaline buffer (pH 10.5). A control sample contained only BBMVs. The reaction was stopped with 1 mM PMSF and the BBMVs were recovered by centrifugation at 157000 ***g*** for 30 min at 4°C. The pellet was washed once with 100 μl of alkaline buffer, and finally suspended in 60 μl of the same buffer. Then, 4× Laemmli sample buffer was added and the sample was divided into three tubes, which were incubated for 3 min at different temperatures as described above. After heating, these samples were separated by SDS/PAGE (8% gels), electrotransferred on to PVDF membranes and revealed by Western blot assays as described below.

### Western blot assays

PVDF membranes were blocked with 5% (w/v) non-fat dried skimmed milk powder in washing buffer (PBS, pH 7.4, plus 0.1% Tween 20), for 1 h at room temperature. The membranes were rinsed once with the same buffer. Cry1Ab was detected with polyclonal anti-Cry1Ab antibodies and the cadherin receptor protein was detected with polyclonal anti-cadherin antibodies (1/30000 dilution; 1 h). Visualization was performed with goat anti-rabbit secondary antibody coupled to horseradish peroxidase (Santa Cruz Biotechnology) (1/25000 dilution; 1 h), followed by SuperSignal™ chemiluminescence substrate (Pierce), according to the manufacturer's instructions.

Both the anti-Cry1Ab and anti-cadherin antibodies were produced in New Zealand white rabbits that were immunized subcutaneously. For the anti-Cry1Ab antibodies, a mixture of oligomeric and monomeric Cry1Ab structures were used for immunization. These structures were obtained after proteolytic activation of Cry1Ab protoxin in the presence of scFv73 antibody, which mimics one of the binding sites of the cadherin receptor [35]. For anti-cadherin antibodies, the cadherin fragment produced in *E. coli* was used for immunization [26]. The rabbits were boosted three times with 1 mg of the Cry1Ab monomeric/oligomeric mixture or with the cadherin fragment, each mixed with incomplete Freund's adjuvant, at 15-day intervals. All procedures involving animals were conducted according to the ethical guidelines of the Instituto de Biotecnología, Universidad Nacional Autónoma de México.

### Planar lipid bilayer experiments

For pore-formation assays, the SUV membrane fractions containing Cry1Ab oligomers were suspended in 100 μl of 10 mM Ches and 300 mM KCl (pH 9). These oligomers were formed after interaction of trypsin-activated Cry1Ab toxin or protoxin with the cadherin fragment in the presence of trypsin as reported above in the ‘Incorporation of oligomers into SUVs (small unilamellar vesicles)’ section. PLBs were made using the method of Müeller et al. [36] with DPPC (1,2-dipalmitoyl-*sn*-glycero-3-phosphocholine) (Avanti Polar Lipids) (20 mg/ml in n-decane). Typical bilayer capacitance values were between 250 and 350 pF. Buffer solution (10 mM Ches and 300 mM KCl, pH 9) was added to the *cis* compartment, whereas, in the *trans* compartment, the buffer solution was modified to contain 150 mM KCl. Different KCl concentrations in the *cis* and *trans* compartments facilitate fusion of membrane vesicles to the bilayer by adding the vesicle suspension containing Cry1Ab protein samples to the *cis* compartment, whereas the *trans* was held at virtual ground. All experiments were performed at room temperature. Ionic currents were recorded with a Dagan 3900A patch-clamp amplifier. Currents were filtered at 200 or 500 Hz and digitized online at 1 or 2 kHz respectively, and analysed on a personal computer using a Digidata 1200 interface and Axotape and pClamp software (Axon Instruments). Histograms with the most frequently observed currents were constructed with pSTAT program version 6.0.5 using Simplex SLQ fitting. The open probability (*P*_o_) was calculated by (*t*_o_/*t*_i_)/*N*, where *t*_o_ represents the sum of open times by *N* channels with different open levels over the total recording time interval (*t*_i_).

### Association and dissociation binding assays of Cry1Ab toxins to BBMVs

For binding association assays, 2 nM activated Cry1Ab toxin was incubated with 10 μg of BBMVs for 0, 20, 40, 60 and 100 min at 30°C in binding buffer (PBS and 0.1% BSA, pH 10.5) with slow agitation. After 100 min of binding, the time-dependent dissociation process was analysed after dilution of the mixture with 20-fold volumes of the same buffer. The unbound toxin was removed by centrifugation at ~18500 ***g*** for 10 min and the membrane pellets containing the bound toxin were washed once with same buffer, suspended in 10 μl of PBS and 3 μl of 4× Laemmli sample buffer and heated for 5 min at 50 or 100°C. These samples were separated by SDS/PAGE (8% gels) and electrotransferred on to PVDF membranes. Proteins were revealed in Western blot assays using rabbit anti-Cry1Ab antibodies as described above.

### Toxicity assays against *M. sexta* larvae

Bioassays were performed with *M. sexta* neonatal larvae. Different doses of activated toxin or protoxin solutions were poured on to the diet surface in 24-well polystyrene plates and allowed to dry. A total of 24 neonatal *M. sexta* larvae per plate per dose were analysed in triplicate, and five different doses were assayed. Mortality was monitored after 7 days and the LC_50_ (50% lethal concentration) was analysed using Probit (LeOra Software).

## RESULTS

### Analysis of the binding interaction of Cry1Ab protoxin or toxin with cadherin receptor

**Figure 1:**
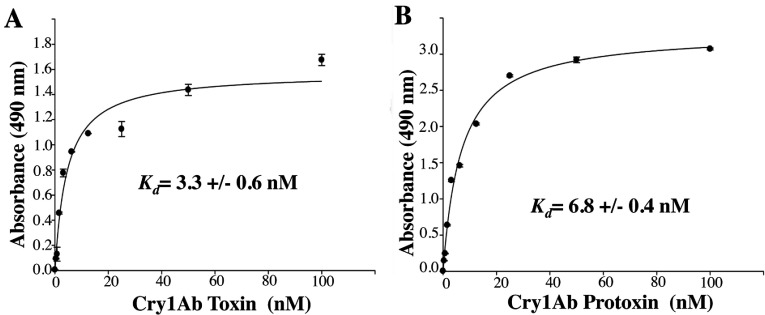
Binding of Cry1Ab toxin or protoxin to a cadherin fragment (CR7–CR12) from *M. sexta* larvae ELISA saturation binding assays were performed by fixing 1 μg of cadherin fragment per well in ELISA plates and then incubating with different concentrations of pure activated Cry1Ab toxin (**A**) or Cry1Ab protoxin (**B**). Bound Cry protein was revealed with polyclonal anti-Cry1Ab antibody. Data were analysed using Scatchard analysis with SigmaPlot.

Interaction with the cadherin receptor is a crucial step in the mode of action of Cry toxins. It was shown previously that Cry1Ac protoxin can bind *Pectinophora gossypiella* cadherin [37]. However, the kinetics of this binding was not determined. In the present study, we analysed, using ELISA saturation binding assays, the binding of Cry1Ab protoxin or activated Cry1Ab toxin with the *M. sexta* cadherin fragment, which contains the three known toxin-binding sites (CR7, CR11 and CR12) [26]. We found that Cry1Ab protoxin binds to the cadherin receptor fragment with high affinity (Figure 1). The apparent dissociation constant (*K*_d_) of the Cry1Ab protoxin–CR7–CR12 interaction (6.8±0.4 nM) was similar to that of the Cry1Ab toxin–CR7–CR12 interaction (3.3±0.6 nM), suggesting that, *in vivo*, both the protoxin and the activated monomeric toxin would be able to bind to the cadherin receptor.

### Oligomerization of Cry1Ab toxin or protoxin in solution, after interaction with cadherin receptor

**Figure 2:**
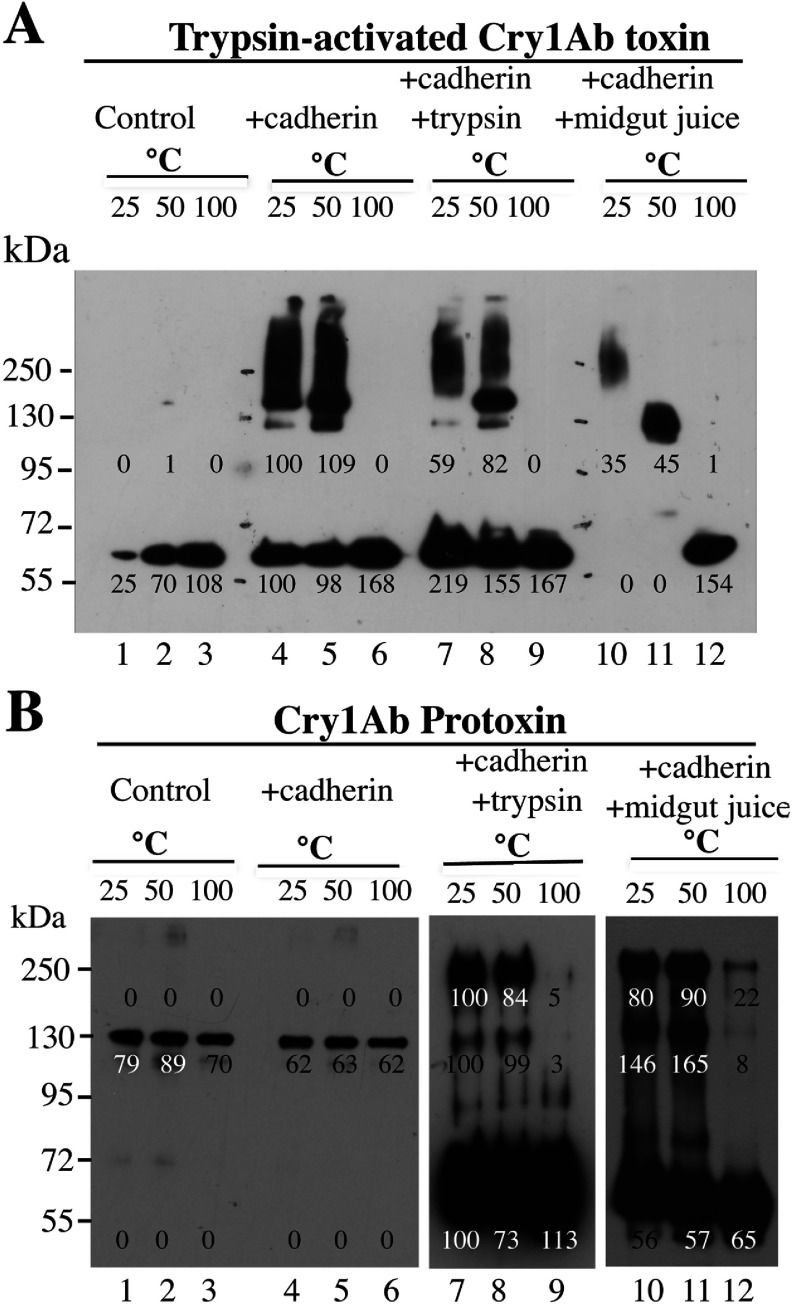
Oligomerization in solution of Cry1Ab toxin or protoxin, after interaction with a cadherin fragment from *M. sexta* Monomeric trypsin-activated Cry1Ab toxin (**A**) or protoxin (**B**) were incubated in solution at 37°C for 1 h with the cadherin CR7–CR12 fragment in the presence or absence of trypsin or midgut juice proteases from *M. sexta*. After incubation, samples in Laemmli sample buffer were heated for 5 min at different temperatures and revealed in Western blot assays with anti-Cry1Ab antibody. Lanes 1–3, control samples of Cry1Ab toxin or protoxin; lanes 4–6, incubation of Cry1Ab toxin or protoxin with the cadherin fragment; lanes 7–9, incubation of Cry1Ab toxin or protoxin with the cadherin fragment in the presence of trypsin; lanes 10–12, incubation of Cry1Ab toxin or protoxin with the cadherin fragment in the presence of midgut juice from *M. sexta* larvae. Molecular-mass markers were PageRuler pre-stained protein ladder (Fermentas) and molecular masses are indicated in kDa. Numbers under the bands represent the percentage of each band on the blot calculated after densitometric analysis of the bands using ImageJ software and using one band of the same mass in the gel as 100% reference.

To determine the role of protoxin binding to cadherin, we compared oligomerization of the Cry1Ab trypsin-activated toxin (Figure 2A) with that of the protoxin (Figure 2B). This comparison was done after interaction with the cadherin fragment from *M. sexta*, in the presence or absence of trypsin or midgut juice proteases. The thermosensitivity of the oligomeric structures was analysed by Western blot assays, incubating the samples at different temperatures (25, 50 or 100°C) for 5 min before SDS/PAGE. In control experiments, the Cry1Ab proteins were also incubated for 5 min at these temperatures, showing that these incubations did not affect their molecular mass as resolved by SDS/PAGE (Figures 2A and 2B, lanes 1–3). We were surprised that incubation of trypsin-activated toxin with cadherin, in the absence of proteases, caused significant changes in the Cry1Ab structure to induce its oligomerization, forming high-molecular-mass structures (150–250 kDa) that were SDS-resistant but temperature-sensitive, since 5 min of incubation at 100°C completely disassembled the oligomeric structures into 60 kDa monomeric toxin (Figure 2A, lanes 4–6). Supplementary Figure S1 (http://www.biochemj.org/bj/459/bj4590383add.htm) shows that these structures were not formed by interaction with other receptors such as APN or ALP. Figure 2(A) (lanes 7–9) shows that similar oligomeric structures were observed if the activated toxin was incubated with the cadherin fragment in the presence of trypsin, suggesting that no further cleavages were induced by trypsin treatment. However, if this incubation was carried out in the presence of proteases from insect midgut, the oligomeric structure that is formed is slightly different, showing a single band of 250 kDa when the sample was heated at 25°C before SDS/PAGE. This oligomeric structure was highly sensitive to temperature, since its mass is reduced to 130 kDa when heated at 50°C and disassembled into monomeric toxin when heated at 100°C before SDS/PAGE (Figure 2A, lanes 10–12). A similar experiment, where the activated Cry1Ab toxin was incubated with cadherin in the presence or absence of proteases, but revealed with an anti-cadherin antibody (Supplementary Figure S2 at http://www.biochemj.org/bj/459/bj4590383add.htm), showed that the cadherin was not part of the oligomeric toxin structures (Supplementary Figure S2, lanes 4–6). Furthermore, the cadherin was cleaved after incubation with trypsin (Supplementary Figure S2, lanes 7–9) or midgut juice (Supplementary Figure S2, lanes 10–12).

In contrast, incubation of Cry1Ab protoxin with the cadherin fragment did not affect its apparent molecular mass of 130 kDa (Figure 2B, lanes 4–6). However, in the presence of trypsin (Figure 2B, lanes 7–9) or midgut juice from *M. sexta* (Figure 2B, lanes 10–12), most of the protoxin is transformed into monomeric toxin, and oligomers of molecular mass close to 250 kDa are produced. These oligomers are stable after heat treatment at 50°C (compare lanes 7 and 8 or lanes 10 and 11 of Figure 2B). It is evident that these structures are also disassembled after incubation at 100°C. However, in the presence of midgut juice, some oligomeric structure still could be observed after incubation at this temperature (Figure 2B, lane 12).

These data indicate that interaction of cadherin facilitates oligomerization of Cry1Ab and that two different oligomeric structures are formed in solution depending on whether the interacting molecule is a trypsin-activated toxin or protoxin.

### Insertion of Cry1Ab pre-pore structures into SUVs or in BBMVs from *M. sexta*

**Figure 3:**
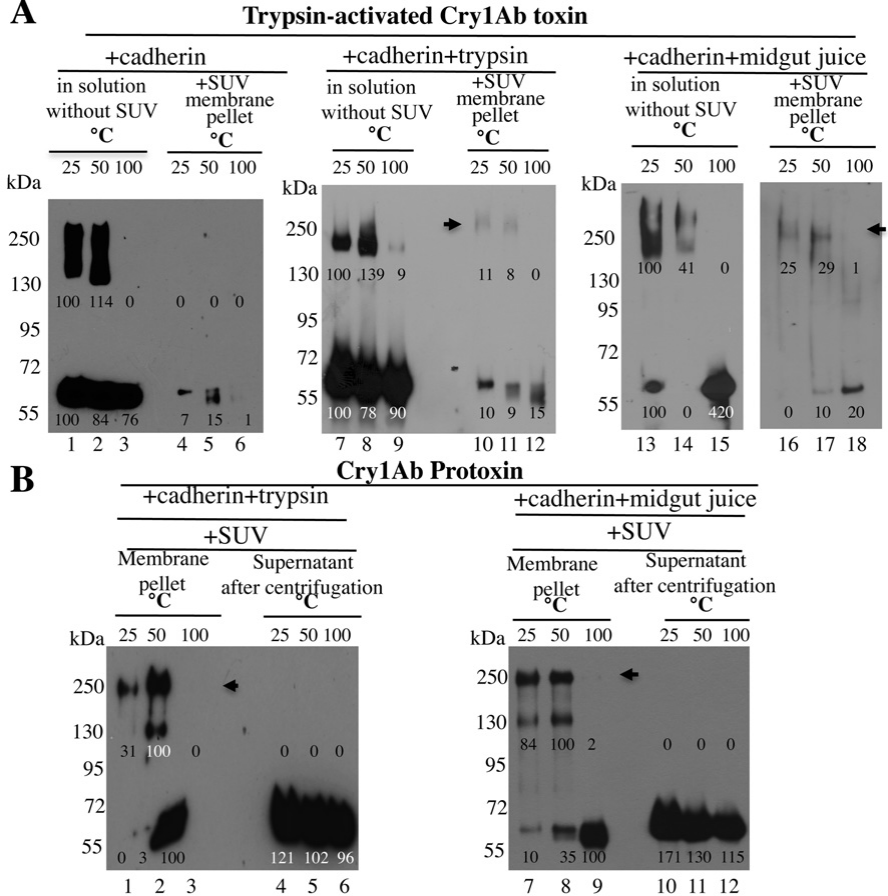
Partition into SUVs of oligomeric structures formed in solution from Cry1Ab toxin or protoxin (**A**) Samples containing oligomeric and monomeric structures of Cry1Ab toxin obtained in solution after incubation with the cadherin fragment (lanes 1–3), with the cadherin fragment in the presence of trypsin (lanes 7–9), or with the cadherin fragment and midgut juice proteases from *M. sexta* (lanes 13–15) at 37°C (as in Figure 2). Samples were incubated for 1 h with SUV liposomes, and finally centrifuged at 157000 ***g*** for 30 min. Pellet samples were recovered (in a total volume of 60 μl of PBS) and divided into three different tubes (20 μl each), Laemmli sample buffer was added, and each was heated for 5 min at a different temperature and revealed in Western blot assays with anti-Cry1Ab antibody. Proteins obtained in pellets represent proteins incorporated into the SUV membrane obtained under the three different conditions (lanes 4–6, 10–12 and 16–18). (**B**) Samples obtained after incubation in solution of Cry1Ab protoxin with the cadherin fragment and trypsin or midgut juice proteases from *M. sexta* at 37°C as in Figure 2 were incubated afterwards for 1 h with SUV liposomes, and finally centrifuged at 157000 ***g*** for 30 min. Pellets (recovered as above in a total volume of 60 μl of PBS were divided into three different tubes) or 20 μl of supernatant samples were used. Laemmli sample buffer was added to each sample, which were each then heated for 5 min at a different temperature before being revealed in Western blot assays with anti-Cry1Ab antibody. Lanes 1–3, proteins obtained in SUV pellets; lanes 4–6, proteins from supernatants after incubation of Cry1Ab protoxin with the cadherin fragment in the presence of trypsin; lanes 7–9, proteins incorporated into membrane pellet; lanes 10–12, proteins in the supernatants from samples obtained after incubation of Cry1Ab protoxin with the cadherin fragment in the presence of midgut juice. Arrows point to the Cry1Ab oligomers incorporated into membrane pellets of SUVs. Numbers under the bands represent the percentage of each band on the blot calculated after densitometric analysis of the bands using ImageJ software and using one band of the same mass in the gel as 100% reference. Molecular masses are indicated in kDa.

To determine whether the observed oligomers are able to insert into the membrane, similar oligomerization assays in solution were performed with trypsin-activated Cry1Ab or Cry1Ab protoxin. These protein mixtures were incubated afterwards for 1 h with SUVs. The membrane pellets, containing proteins inserted into the vesicles, were separated by ultracentrifugation, resolved by SDS/PAGE and revealed by Western blot. Figure 3 shows how the different oligomeric structures partition into the SUV membrane. First, when the activated Cry1Ab toxin was incubated in solution with cadherin in the absence (Figure 3A, lanes 1–3) or presence (Figure 3A, lanes 7–9) of trypsin, the oligomer that is formed in solution did not insert efficiently into the SUVs (Figure 3A, lanes 4–6 and 10–12 respectively). A small amount of toxin could be detected in the membrane pellet, which could represent some protein precipitation, since it was not observed at the three temperatures (Figure 3A, lanes 4–6). The oligomer obtained after the Cry1Ab toxin was incubated in solution with cadherin in the presence of midgut juice (Figure 3A, lanes 13–15) was partially able to insert into the liposome (Figure 3A, lanes 16–18, arrow).

In contrast, the 250-kDa oligomeric structure obtained after incubation of Cry1Ab protoxin with cadherin in the presence of trypsin (Figure 3B, lanes 1–6) or midgut juice (Figure 3B, lanes 7–12) was able to insert into SUVs (Figure 3B lanes 1–3 and 7–9 respectively, arrow), and the supernatant only contained the monomeric toxin (Figure 3B, lanes 4–6 and 10–12). A control of the different toxin and protoxin incubations without liposomes showed an absence of protein precipitation (results not shown).

**Figure 4:**
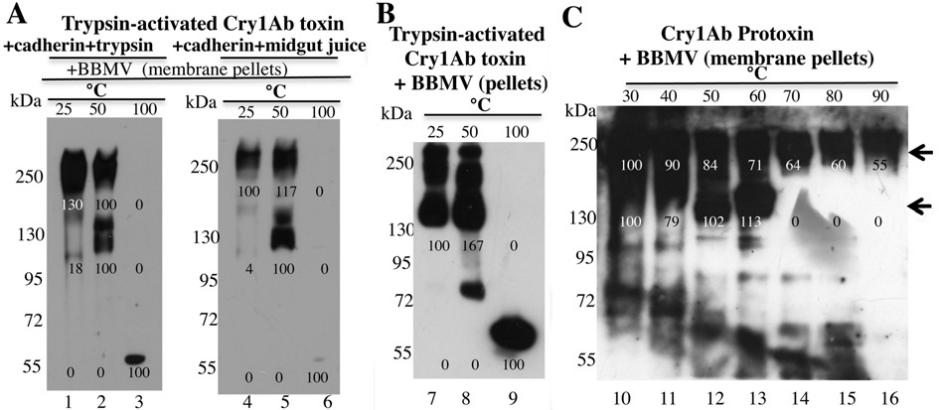
Partitioning into BBMVs isolated from *M. sexta* larvae of oligomers obtained previously in solution and oligomerization of Cry1Ab toxin or protoxin after direct interaction with BBMVs (**A**) BBMV incorporation of oligomers previously formed in solution after incubation of Cry1Ab toxin with the cadherin fragment in the presence of trypsin (lanes 1–3) or midgut juice from *M. sexta* (lanes 4–6). Protein samples obtained in solution were incubated for 1 h with BBMVs, and finally centrifuged at 157000 ***g*** for 30 min. (**B** and **C**) Oligomer formation after direct interaction with BBMVs. Cry1Ab toxin (**B**) or Cry1Ab protoxin (**C**) were incubated at 37°C for 1 h with BBMVs. After incubation, the samples were centrifuged at 157000 ***g*** for 30 min. The pellet samples were washed with buffer by centrifugation and finally suspended in Laemmli sample buffer and each was heated for 3 min at a different temperature before resolving by SDS/PAGE and Western blot assay using anti-Cry1Ab antibody. Arrows point to the two types of oligomers that are formed: a 150–250-kDa heat-sensitive oligomer (lanes 10–13) and a 250-kDa oligomer that is more resistant to heat treatment (lanes 10–16). Numbers under the bands represent the percentage of each band on the blot calculated after densitometric analysis of the bands using ImageJ software and using one band of a similar mass in the gel as 100% reference. Molecular masses are indicated in kDa.

It is important to note that oligomeric structures formed in solution from activated toxin (Figure 3A, lanes 7–9 and 13–15) were able to insert efficiently into BBMVs isolated from *M. sexta* as shown in Figure 4(A). The oligomers were recovered in the BBMV pellet, indicating that the presence of receptors is important for insertion of these oligomeric structures into the membrane, supporting the sequential binding model.

We also analysed the oligomerization of Cry1Ab toxin or protoxin after their direct interaction with BBMVs isolated from *M. sexta*, which contain all toxin receptors. Figure 4(B) shows that when activated Cry1Ab toxin was incubated with BBMVs, heat-sensitive 150–250-kDa oligomeric structures were observed associated with the membrane pellet. If the Cry1Ab protoxin was incubated with BBMVs (Figure 4C), two different oligomeric structures (arrows) could be observed associated with the membrane pellet, a 150–250-kDa heat-sensitive oligomer (Figure 4C, lanes 10–13) and a 250-kDa oligomer that is more resistant to heat treatment (Figure 4C, lanes 10–16). These results show that both pre-pore oligomeric structures are able to insert into BBMVs.

### Pore-formation activity

**Figure 5:**
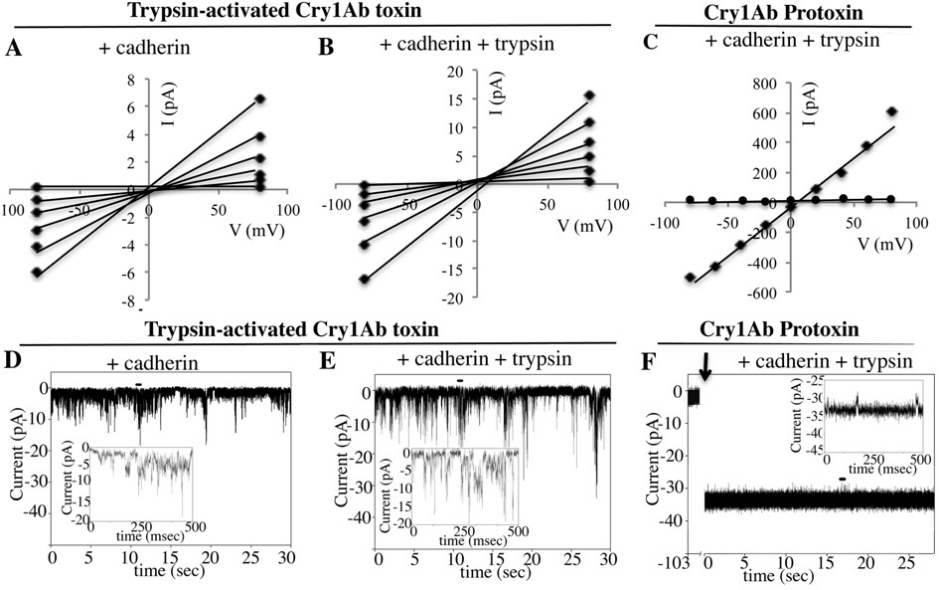
Pore-formation activity in black lipid bilayers Current–voltage (*I*–*V*) relationship of the currents induced by activated Cry1Ab toxin (**A** and **B**) or Cry1Ab protoxin (**C**) after interaction with the cadherin fragment (**A**) or with cadherin fragment plus trypsin (**B** and **C**) and recovered in SUV liposomes as described in the Experimental section. Representative ionic pore records of most common transitions induced by these samples are shown in (**D**) and (**E**) for oligomers obtained from activated toxin (after incubation with cadherin or cadherin plus trypsin respectively) and (**F**) from Cry1Ab protoxin (after incubation with cadherin plus trypsin). Records were obtained in asymmetrical 300 mM *cis* and 150 mM *trans* KCl conditions. Insets in (**D**)–(**F**) each show a small portion of the recorded pores marked with a small horizontal line over the trace, on a millisecond timescale. Arrow in (**F**) indicates SUV liposome addition to the *cis* bilayer chamber. ◆, data after SUV addition; ●, data before SUV addition.

To determine whether oligomers formed from activated toxin or protoxin are able to induce pore formation, we analysed the pore-formation activity of the membrane-associated proteins using voltage clamp on PLBs as described in the Experimental section. Figures 5(A)–5(C) show current–voltage (*I*–*V*) relationships of the induced currents by the different samples. Figures 5(D)–5(F) show 30-s records of the pores induced by these samples. Liposomes with associated Cry proteins were fused to PLBs before the pore-formation activity was analysed [25–27,33]. The samples obtained from activated Cry1Ab toxin formed ionic pores that were similar to the pores induced by monomeric toxin reported previously [14,16–20], showing pore activity with multiple subconducting states that are difficult to resolve and with low open probability with values of 0.3–0.6 (Figures 5A, 5B, 5D and 5E). To analyse these currents, histograms with the most common ionic currents at −80 mV and at 80 mV were constructed and these data were plotted as an *I*–*V* graph (Figures 5A and 5B). The oligomer that is formed after incubation of activated toxin with cadherin and midgut juice induces macroscopic currents with multiple subconducting states, probably due to insertion of multiple pores (results not shown). In contrast, the oligomer obtained from Cry1Ab protoxin incubated with cadherin and trypsin induces the formation of highly stable ion pores with high open probability (*P*_o_ close to 1) (Figures 5C and 5F), similar to the previously reported pores induced by pure oligomeric structures obtained from Cry1Ab protoxin activated with midgut juice in the presence of svFv73 antibody which mimics cadherin receptor [16,25–27]. The higher current in the samples obtained with Cry1Ab protoxin could be due to insertion of several pores in the same liposome that fuses to the lipid bilayer. The samples obtained after interaction of Cry1Ab protoxin with cadherin and midgut juice also induced macroscopic currents with stable open probability probably due to the insertion of multiple pores (results not shown).

### Irreversible binding of Cry1Ab monomer to BBMVs is due to the membrane insertion of the heat-sensitive oligomer

Association and dissociation binding assays of Cry1Ab toxin to *M. sexta* BBMVs were performed to study the reversible and irreversible binding components of the interaction of Cry1Ab toxin with BBMVs. We analysed the time-dependent association of Cry1Ab toxin with BBMVs up to 100 min. After this period, the time-dependent dissociation process was determined up to 60 min, by diluting the mixture with 20-fold volumes of buffer. The samples were centrifuged, and toxin bound to BBMVs was visualized by Western blotting. Total binding corresponds to the toxin bound after 100 min of association, and the irreversible binding was determined as the bound toxin that remains after 60 min under dissociation conditions.

**Figure 6:**
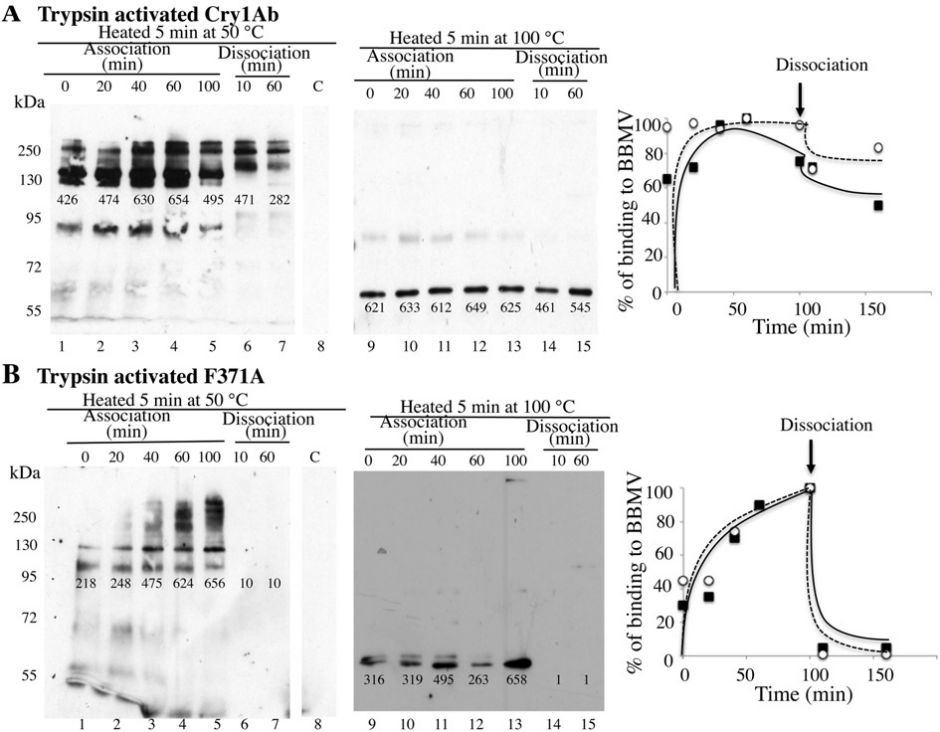
Association and dissociation binding assays of Cry1Ab or F371A toxins to BBMVs isolated from *M. sexta* larvae (**A**) Time-dependent association of monomeric Cry1Ab toxin to BBMVs was analysed for up to 100 min (lanes 1–5 and 9–13). After this time, the sample was diluted by addition of an excess of 20-fold volume of buffer and time-dependent dissociation was analysed for up to 60 min (lanes 6 and 7, and 14 and 15). Unbound toxin was removed by centrifugation and the membrane pellet containing the bound toxin was analysed by Western blot assay with anti-Cry1Ab antibody. Samples in Laemmli sample buffer were heated at 50°C (lanes 1–8) or at 100°C (lanes 9–15) for 5 min before SDS/PAGE and visualizing the proteins in Western blot assays. Lane 8 is control BBMVs without toxin incubation. Molecular masses are indicated in kDa. (**B**) Similar experiment to that described in (**A**) performed with monomeric F371A mutant toxin. Densitometric analysis of the bands in the blot was carried out using ImageJ software to quantify binding. These values were plotted as the percentage of binding against time. Closed symbols correspond to data of blots obtained after heating at 50°C and open symbols correspond to data of blots obtained after heating at 100°C.

To detect the oligomeric structure of the Cry1Ab toxin, we heated these samples for 5 min at 50°C before resolving by SDS/PAGE (Figure 6A, lanes 1–8). These samples were also heated at 100°C (Figure 6A, lanes 9–15). Figure 6(A) shows that when Cry1Ab toxin is bound to BBMVs, an oligomeric structure of 150–250 kDa was formed and it was incorporated into the membranes as an SDS-resistant heat-sensitive oligomer (Figure 6A, lanes 1–5) that disassembled if samples were heated at 100°C (Figure 6A, lanes 9–13). The dissociation assays indicate that these oligomeric structures were not washed out after dilution of the sample with a 20-fold higher amount of buffer, suggesting a stable insertion of oligomeric molecules into the membrane (Figure 6A, lanes 6 and 7, and 14 and 15). The irreversible Cry1Ab binding represented 57% of the total toxin binding, as determined by densitometric analysis of the bands (at 50°C) using ImageJ software (NIH). A similar experiment was performed with biotinylated Cry1Ab toxin, and bound toxin was visualized with streptavidin coupled to peroxidase. In this case, competition was carried out with a 500-fold higher concentration of unlabelled toxin. Supplementary Figure S3 (http://www.biochemj.org/bj/459/bj4590383add.htm) shows that Cry1Ab bound BBMVs in an irreversible way and, after competition with unlabelled toxin, 59% of the toxin remained bound to the BBMVs (Supplementary Figure S3). If the sample was heated at 100°C before loading the gels, a monomeric band was observed (Supplementary Figure S3, lanes 9–14), such as shown previously in multiple binding assays performed with biotinylated toxins [11,25,26,35]. However, if samples were heated at 50°C, no band was observed (Supplementary Figure S3, lanes 1–6), indicating that the biotin molecule is not accessible to the streptavidin, probably because it is buried within the oligomeric toxin structure, which was clearly observed if the blots were revealed with anti-Cry1Ab antibody (Figure 6A, lanes 1–7).

The Cry1Ab F371A mutant that is affected in its irreversible binding to BBMVs [28,38] was used as a control in these experiments. Heat-sensitive oligomers were observed when trypsin-activated F371A mutant was incubated with BBMVs (Figure 6B, lanes 1–5 and lanes 9–13). Nevertheless, binding of these oligomeric structures is highly reversible since all binding was lost during the dissociation assay (Figure 6B, lanes 6 and 7, and 14 and 15).

Overall, these data indicate that irreversible binding of trypsin-activated Cry1Ab toxin with BBMVs of *M. sexta* correlates with a stable insertion of the oligomeric structure of the toxin into the membrane. Also, the monomeric toxin was not inserted into the membrane, since oligomeric structures were observed at the shortest time of incubation (*t*_0_). Mutant F371A is not affected in oligomerization, since oligomer formation can be observed in the membrane pellet, but insertion of these oligomers into the membrane is slower and completely reversible. It did not display a stable interaction with BBMVs.

### Characterization of Cry1AbMod toxin

**Figure 7:**
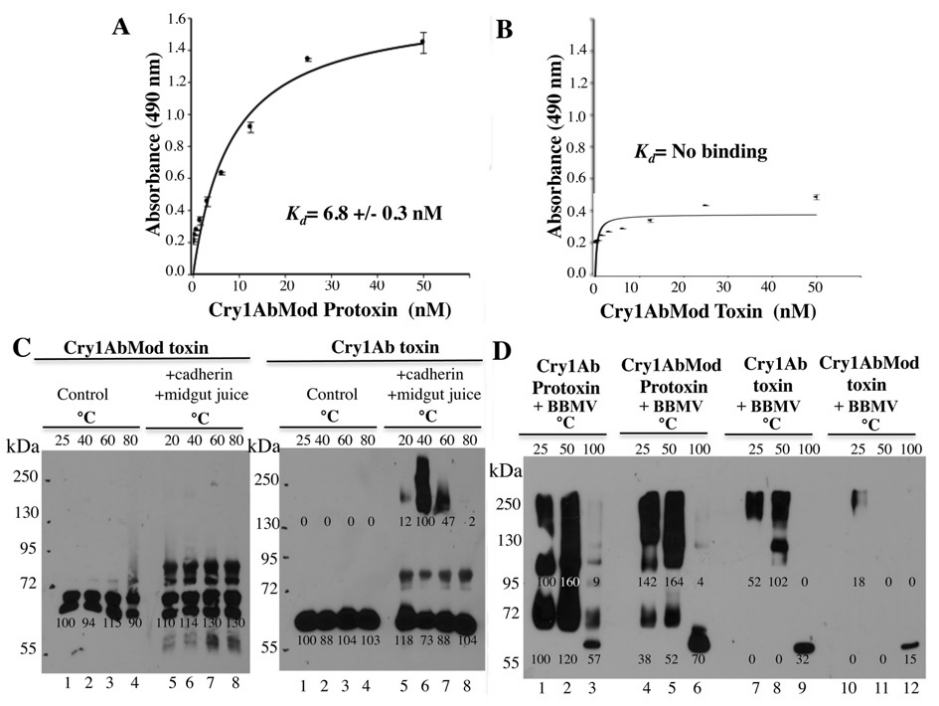
Analysis of binding of Cry1AbMod to the cadherin fragment (CR7–CR12) and oligomerization of Cry1AbMod toxin and protoxin ELISA saturation binding assays were performed by fixing 1 μg of cadherin fragment in ELISA plates and incubating with different concentrations of pure Cry1AbMod protoxin (**A**) or pure trypsin-activated Cry1AbMod toxin (**B**). Bound protein was revealed with polyclonal anti-Cry1Ab antibody. Data were analysed using Scatchard analysis with SigmaPlot. (**C**) Oligomerization in solution of Cry1AbMod or Cry1Ab toxins after interaction with the cadherin fragment, in the presence of midgut juice proteases from *M. sexta* at 37°C. After incubation, each sample in Laemmli sample buffer was heated for 5 min at a different temperature and revealed by Western blot assay with anti-Cry1Ab antibody. Lanes 1–4, control Cry1AbMod toxin or Cry1Ab toxin; lanes 5–8, incubation of Cry1AbMod toxin or Cry1Ab toxin with the cadherin fragment in the presence of midgut juice. (**D**) Oligomerization of Cry1AbMod or Cry1Ab, in their protoxin or activated toxin forms after interaction with BBMVs isolated from *M. sexta* larvae. Lanes 1–3, incubation of Cry1Ab protoxin; lanes 4–6, Cry1AbMod protoxin with BBMVs; lanes 7–9, Cry1Ab toxin; lanes 10–12, Cry1AbMod toxin after incubation for 1 h with BBMVs at 37°C. After incubation, samples were centrifuged at 157000 ***g*** for 30 min. The pellet samples were washed with buffer by centrifugation and finally suspended in Laemmli sample buffer, heated at different temperatures for 3 min and revealed in Western blot assays with anti-Cry1Ab antibody. Numbers under the bands represent the percentage of each band on the blot calculated after densitometric analysis of the bands using ImageJ software and using one band of the same mass in the gel as 100% reference. Molecular masses are indicated in kDa.

Cry1AbMod is a mutant protein where the N-terminal end, including helix α-1 and a small part of helix α-2a, is deleted [29]. We activated the Cry1AbMod protoxin with trypsin and purified the trypsin-activated Cry1AbMod toxin by ion-exchange chromatography. Analyses of Cry1AbMod toxin and protoxin binding to the cadherin fragment CR7–CR12 show that the Cry1AbMod protoxin was able to bind the cadherin fragment with a similar affinity as that of the Cry1Ab protoxin (Figure 7A), whereas the activated Cry1AbMod toxin lost its ability to bind cadherin (Figure 7B).

It was reported that the Cry1AbMod protoxin forms a 250-kDa oligomeric structure after trypsin activation in the absence of cadherin. This structure is resistant to temperature and inserts into synthetic liposomes [27,29]. Furthermore, the Cry1AbMod protoxin oligomerizes more efficiently at high alkaline pH and in the presence of SUV liposomes than in solution [27]. Thus we analysed the oligomerization of the ion-exchange-purified trypsin-activated Cry1AbMod toxin, showing that it was unable to oligomerize in solution after midgut juice treatment (Figure 7C). Oligomerization of Cry1AbMod was also analysed in the presence of BBMVs and we found that the Cry1AbMod protoxin formed oligomers that insert into BBMVs. In contrast, the activated Cry1AbMod toxin oligomerized and inserted into BBMVs less efficiently (Figure 7D).

The insecticidal activity of the protoxin and the activated Cry1AbMod toxin against *M. sexta* larvae were analysed. Table 1 shows that Cry1AbMod toxin had 8-fold lower insecticidal activity than the Cry1AbMod protoxin. In contrast, both wild-type Cry1Ab toxin or protoxin have similar insecticidal activities against *M. sexta* (Table 1). These data could explain why Cry1AbMod lost its potency against certain susceptible larvae [29,39,40], since the activated toxin results in a protein with reduced oligomerization and is unable to bind cadherin. These data suggest that participation of the activated toxin is important for insecticidal activity against susceptible larvae.

**Table 1:** Toxicity of Cry1Ab and Cry1AbMod against *M. sexta* larvae LC_50_, 50% lethal concentration, analysed statistically with Probit software.

Protein	LC_50_ (ng/cm^2^) (90% confidence limits)
Cry1Ab protoxin	1 (0.5–1.7)
Cry1Ab toxin	2 (1.6–2.7)
Cry1AbMod protoxin	2 (1.3–3.0)
Cry1AbMod toxin	15 (11.2–18.3)

## DISCUSSION

### Oligomerization of Cry toxins takes place outside the membrane and the induced oligomer in solution is similar to the oligomer that is formed in the presence of BBMVs

Our data indicate that two distinct Cry1Ab pre-pore structures are formed in solution after cadherin binding, depending on the Cry protein involved, whether it is the trypsin-activated toxin or the protoxin. Both the activated toxin and protoxin bind the cadherin fragment CR7–CR12 with high affinity from *M. sexta* larvae (Figure 1). The interaction of Cry1Ab toxin with the cadherin fragment is sufficient to induce the formation of 150–250-kDa oligomeric structures in solution, and no extra cleavage is needed for the formation of these oligomers (Figure 2A). Trypsin treatment did not affect the mass of these oligomers (Figure 2A). However, these oligomers do not insert into synthetic liposomes efficiently (Figure 3A) and form pores with multiple subconducting states and low open probability (Figures 5A, 5B, 5D and 5E). Midgut juice treatment after interaction of Cry1Ab toxin with cadherin induces a change in the mass of the oligomers (Figure 2A), and these structures show slightly better insertion into synthetic membranes (Figure 3A). Importantly, oligomers that were formed after interaction of Cry1Ab toxin with cadherin readily insert into BBMVs (Figure 4A). Thus pre-pore structures formed outside the bilayer require other proteins to insert efficiently into the membrane, such as has been proposed previously for APN or ALP receptors [8,9], supporting the sequential binding model of toxin pore formation [12].

In contrast, incubation of protoxin with the cadherin fragment and midgut juice proteases or trypsin in solution induces the formation of a 250-kDa oligomer that inserts into synthetic liposomes, as demonstrated previously [16,25–27]. Analyses of pore-formation activity in black lipid bilayers of these oligomeric structures showed the induction of stable ion pores with high open probability (Figures 5C and 5F) as reported previously [16,25–27].

An important conclusion of the present study is that interaction with cadherin receptor is required to induce oligomerization of Cry1Ab toxin or protoxin. Incubation with other receptors such as those for APN and ALP did not induce Cry1Ab oligomerization (Supplementary Figure S1), but these other receptors may be important for insertion of Cry1Ab oligomers into BBMVs (Figure 4A). Both oligomeric structures were SDS-resistant and heat-sensitive. However, the 250-kDa oligomer obtained from Cry1Ab protoxin was shown to be more heat-resistant, since part of it could be observed even after heating the samples at 100°C (Figures 2B and 4C). Importantly, the formation of this structure *in vitro* seems to be less efficient, since it was less abundant than the oligomeric structure observed after interaction of Cry1Ab toxin with cadherin (Figure 2). This may be due to degradation of the cadherin fragment by midgut juice proteases as shown in Supplementary Figure S2.

Oligomerization of Cry1Ab toxin or protoxin in the presence of BBMVs was also analysed. Cry1Ab toxin induces the formation of oligomeric structures of 150–250 kDa that are highly sensitive to heat, similar to those that are produced in solution (Figure 4B). In contrast, the Cry1Ab protoxin induces the formation of two oligomeric structures (Figure 4C): one that is highly sensitive to heat and the other of 250 kDa that is more resistant to heat. The latter is similar to those that are produced in solution after protoxin interaction with cadherin and midgut juice protease treatment (Figure 2). In the literature, there are multiple examples that show that pore-formation activity of Cry toxins is highly improved in the presence of BBMVs from target insects such as *M. sexta* [16,41], *Lymantria dispar* [20], *Spodoptera frugiperda* [42] and *Heliothis virescens* [43] or in the presence of aminopeptidases and ALPs from *M. sexta* [44]. These data suggest that the presence of receptors in the BBMVs is essential for Cry toxin action by improving their pore-formation activity. We propose that cadherin induces pre-pore formation and that APN and ALP facilitate its insertion into the membrane. In the present study, we have shown that interaction of Cry1Ab toxin or protoxin with BBMVs results in the formation of oligomeric structures that insert into the membrane. The monomeric toxin is not observed in the BBMV membrane pellet, suggesting that monomeric Cry1Ab toxin does not insert into the target membrane (Figures 4 and 6).

It was shown previously that Cry1Aa toxin, which is highly related to Cry1Ab, also formed oligomeric structures in solution when it is incubated in the presence of a fragment from *Bombyx mori* cadherin BtR-175 containing the Cry1Aa-binding sites, forming a heat-sensitive oligomer similar to that obtained in the presence of *B. mori* BBMVs [45]. In addition, other studies have shown oligomerization of different trypsin-activated Cry1A toxins after incubation with BBMVs isolated from different susceptible insects [46–49]. These structures are inserted into the membrane, have molecular masses higher than 200 kDa and were heat-sensitive, being dissociated into monomeric structures if they were heated at temperatures higher than 70°C [46–48]. It is important to mention that all reports of oligomerization of Cry toxins involve interactions with cadherin or with BBMVs from susceptible insects that contain receptor molecules [12,13,46–50]. These reports are in agreement with the results of the present study which show that interaction with cadherin is a key step that induces oligomerization of Cry1Ab toxin. Further evidence that supports the importance of the cadherin receptor for toxin oligomerization is that a fragment of the *M. sexta* cadherin containing the Cry1Ab-binding sites increases the insecticidal activity of Cry1Ab when fed together with the toxin to different larvae [51,52]. This enhancement in toxicity of Cry toxin by this cadherin fragment was shown to correlate with Cry1Ab oligomer formation [53]. In addition, cadherin fragments isolated from different insect orders such as dipterans or coleopterans also enhanced toxicity of specific Cry toxins active against these insect orders [54,55].

The only exceptions of Cry toxin oligomerization without receptors are mutant toxins that are cleaved between helices α-2a and α-2b or have helices α-1 and α-2a deleted [27,29,45,56–59]. One example is Cry1Aa mutant S373C, which forms heat-sensitive oligomers in solution in the absence of receptors or BBMVs. This mutant is cleaved in the loop between helices α-2a and α-2b after activation, resulting in the loss of helices α-1 and α-2a [45]. Another example is the Cry1AMod protoxins, where helix α-1 and part of helix α-2 are deleted by genetic engineering and these are able to form 250-kDa oligomers in the absence of cadherin interaction [27,29]. The third example is Cry4Ba protein, which is a special case, since it is a native protoxin that is capable of forming toxin oligomers when it is proteolytically activated in the presence of liposomes and in the absence of a cadherin receptor [56–58]. Pore-formation assays in PLBs showed that Cry4Ba oligomers were proficient in forming ion pores [56]. The crystal structure of Cry4Ba showed that, during activation of Cry4Ba protoxin with chymotrypsin, helices α-1 and α-2a were removed and the cleaved protein was crystallized in a trimeric array [58]. Similar results were reported after crystallization of Cry5Ba, showing a cleavage in the corresponding amino acid after helix α-2a and a similar organization of a trimeric array in its crystal structure [59]. It is relevant to mention that silencing experiments of cadherin expression in *Aedes aegypti* by RNAi showed that Cry4Ba does not rely on cadherin molecules for toxicity [56]. All of these data show that cleavage of Cry protoxins in the region between helices α-1 and α-2a is important to induce their oligomerization in the absence of toxin receptors. However, the present study has shown that, *in vitro*, the interaction of activated Cry1Ab toxin with cadherin is enough to form a pre-pore oligomeric structure. But these oligomers require other receptors to insert efficiently into the membrane, since insertion into BBMVs is highly efficient when compared with insertion into synthetic liposomes where insertion is inefficient (compare Figures 3A and 4A).

### Monomeric Cry toxins did not insert into BBMVs and irreversible binding of Cry toxins to BBMVs correlated with stable insertion of oligomer structures into the membrane

It has been argued that irreversible binding of different Cry toxins to BBMVs indicate that a monomeric structure of these toxins was able to insert into the membrane [14,28]. We analysed the kinetics of association and dissociation of Cry1Ab toxin with *M. sexta* BBMVs. Dissociation assays have been used to determine the amount of toxin that remains irreversibly associated with BBMVs [28,60]. Using such assays, we found that binding of Cry1Ab toxin with BBMVs correlates with the formation of a heat-sensitive oligomer, and that irreversible binding to BBMVs correlated with the presence of this structure associated with the membrane pellet. These data are supported by the analysis of irreversible binding of Cry1Aa toxin to *B. mori* BBMVs, where it was shown that irreversible binding is due to the insertion of a heat-sensitive oligomeric structure into the membrane [47].

As a control in our experiments, we analysed the binding of the non-toxic Cry1Ab F371A mutant that is affected in its irreversible binding to BBMVs [28]. It was reported that the loss of irreversible binding of this mutant is due to a lack of toxin insertion into the membrane [28,61]. It was also shown that the monomeric structure of this mutant was able to bind APN and ALP with a similar affinity as did the wild-type toxin. In contrast, the oligomeric form of this mutant was severely affected in binding to these receptors [9]. In the present study, we have shown that F371A toxin binding to BBMVs is correlated with the formation of a heat-sensitive oligomer. This binding was completely dissociated after dilution with buffer, demonstrating further that the irreversible component of Cry toxin binding to BBMVs is due to the stable insertion of the oligomer into the membrane. These data suggest that interaction of the oligomeric structure with APN and ALP receptors is important for insertion of the pre-pore into the membrane, as proposed previously [9,62]. Such evidence supports the sequential binding model of toxin pore formation. In this model, a pre-pore oligomer structure is formed after Cry1Ab interaction with cadherin, and binding of this oligomer to APN and ALP is necessary for its membrane insertion in midgut epithelium cells.

### Both oligomeric structures are functional

In the present study, we provide evidence that the different pre-pore oligomer structures formed after Cry1Ab toxin or protoxin interaction with cadherin have pore-formation activity in black lipid bilayers (Figure 5).

We also show that, in contrast with Cry1Ab and Cry1AbMod protoxins, the activated Cry1AbMod toxin is affected in oligomerization (Figures 7C and 7D). This effect in oligomerization correlated with a 8-fold reduction in insecticidal activity of Cry1AbMod toxin to *M. sexta* larvae, providing evidence that both oligomeric structures are functional and play a role in the intoxication process (Table 1).

On the basis of the proposed role of cadherin in oligomerization of the Cry toxin, it was predicted that Cry1AMod protoxins would counter resistance linked to mutations affecting cadherin expression [29]. Surprisingly, Cry1AMod protoxins have been shown to counter resistance in seven different lepidopteran insects with different mechanisms of resistance, including mutations linked to ABCC2 (ATP-binding cassette C2) transporter, aminopeptidase P genes or to low APN expression [29,39,40]. In the case of the ABCC2 transporter, it has been suggested that it may have a role in facilitating oligomer insertion into the membrane [63]. In the present study, we have shown that insertion of the pre-pore oligomer formed from Cry1Ab protoxin inserts readily into synthetic membranes (Figure 3B), suggesting that this pre-pore has no further need of additional receptor molecules to insert into the membrane. Such data could explain why the Cry1AMod protoxins can counter different resistance mechanisms including mutations in other receptors that might be involved in toxin oligomer membrane insertion.

It has also been shown that, in some lepidopteran species analysed, Cry1AMod protoxins lost potency against susceptible insects [29,39,40], although, in some other insects, the loss in potency of Cry1AbMod is not significant [27,39]. Surprisingly, activated Cry1AbMod toxin lost its affinity for cadherin, whereas the Cry1AbMod protoxin retained its high affinity for the CR7–CR12 fragment (Figures 7A and 7B). It was suggested previously that Cry1Ac activation could occur either before or after binding of protoxin to cadherin and that binding of activated toxin or protoxin could affect its toxicity [37]. Another study suggested that interaction of activated Cry1Ab toxin with cadherin triggered a cascade of intracellular events resulting in necrotic cell death [64]. Therefore we cannot exclude the possibility that the loss of potency of Cry1AMod protoxins against susceptible insects could be related to the loss of a signal transduction component since monomeric Cry1AbMod toxin is affected on cadherin binding. In this regard, a recent observation showed that resistance of a *Helicoverpa armigera* population to Cry1Ac-expressing transgenic cotton in China is linked to a cadherin allele with mutations in the cytoplasmic domain of cadherin [65]. These data suggest that signal transduction mechanisms could be involved in Cry1Ac toxicity at least in this insect species [65]. Nevertheless, the fact that activated Cry1AbMod toxin was less efficient at oligomerization than Cry1AbMod protoxin, may also indicate that the loss of its potency could be due to lack or to the lower amount of this oligomeric structure in the mechanism of action of Cry1AbMod toxin.

Activation of the protoxin to toxin depends on proteases present in each insect species, and this could explain the various levels of Cry1AbMod potency loss in different susceptible insects that have been tested. Potentially, these insects could have different gut proteases that could affect the rate of toxin formation from Cry1AMod protoxin [29,39,40]. Cry1AbMod protoxin shows similar toxicity to native Cry1Ab against some insect pests such as *M. sexta*, *Ostrinia nubilalis*, *P. gossypiella* and *Trichoplusia ni* [27,39]. Nevertheless, Cry1AbMod protoxin was up to 20-fold less toxic against other insects such as *Plutella xylostella* [39]. Notably, in all cases analysed, Cry1AcMod protoxin showed a higher loss of potency compared with Cry1AbMod, such as up to 83-fold less toxicity against *P. gossypiella* [29,39,40]. Such evidence suggests that different processing rates of Cry1AMod protoxins to toxins by midgut proteases in different insect species could correlate with the differential loss of potency of Cry1AMod protoxins against these susceptible lines. Importantly, *Helicoverpa zea* and *H. armigera* populations that are highly resistant to activated Cry1A toxins showed reduced resistance levels to Cry1A protoxins [39,66–68]. Therefore, in these resistant populations, a differential role for the two pre-pores mentioned in the present study may determine the differential susceptibility to Cry proteins.

Most of the PFTs are produced as protoxins, but the role of protoxin fragments in PFT activity has not been analysed. Our data indicate that the protoxin region of PFT molecules may have a functional role that was selected for during evolution, because the presence of this fragment in the protein allows triggering of an alternative mechanism of action that, in some cases, could be quite important [29,39,66–68]. The role of the protoxin region in PFT action was analysed in aerolysin, where it was shown that the C-terminal region that is cleaved after binding to its receptor plays a crucial role for the activity of this PFT by preventing aggregation during biosynthesis and controlling assembly of the quaternary structure of the pre-pore on the membrane surface, functioning as a chaperone [69]. Overall, our data show that Cry toxins are versatile proteins that could exert toxicity by different mechanisms of action and the protoxin molecules trigger the formation of pre-pore structures involved in toxicity. The role of protoxin molecules of other important PFTs in pore formation and virulence remains to be analysed and would be a fruitful area of investigation for the coming years.

## Online data

Supplementary data
