# Supplementary material for: Bacillus thuringiensis Cry1A toxins are versatile proteins with multiple modes of action: two distinct pre-pores are involved in toxicity
Source: Biochem J. 2014 Mar 28;459(Pt 2):383–96. doi: 10.1042/BJ20131408 (PMC3969221; doi:10.1042/BJ20131408)
Supplement: Supplementary data [file bj4590383add.pdf]

Biochem. J. (2014) **459**, 383–396 (Printed in Great Britain) doi:10.1042/BJ20131408

SUPPLEMENTARY ONLINE DATA

Bacillus thuringiensis Cry1A toxins are versatile proteins with multiple modes of action: two distinct pre-pores are involved in toxicity

Isabel GÓMEZ*, Jorge SÁNCHEZ*, Carlos MUÑOZ-GARAY*, Violeta MATUS*, Sarjeet S. GILL†, Mario SOBERÓN*[1] and Alejandra BRAVO*[1]

*Instituto de Biotecnología, Universidad Nacional Autónoma de México, Apdo. Postal 510-3, Cuernavaca 62250, Morelos, Mexico
†Department of Cell Biology and Neuroscience, University of California, Riverside, Riverside, CA 92521, U.S.A.

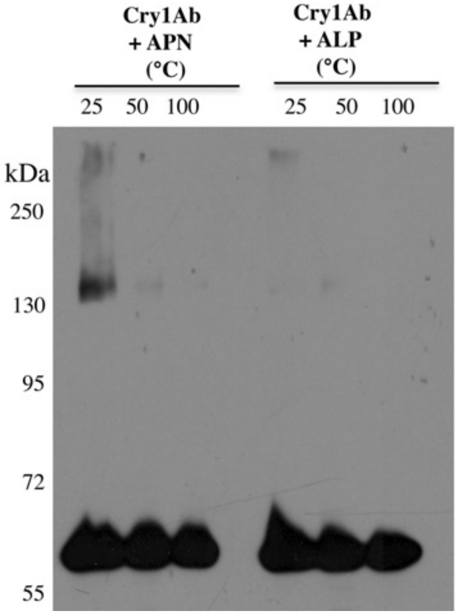

Figure S1 Incubation of Cry1Ab toxin with *M. sexta* APN or ALP proteins in solution

Monomeric Cry1Ab toxin was incubated for 1 h with APN or ALP from *M. sexta* at 37 °C. After incubation, samples were heated for 5 min at different temperatures and revealed in Western blot assays with anti-Cry1Ab antibody. Molecular-mass markers were PageRuler pre-stained protein ladder (Fermentas) and molecular masses are indicated in kDa.

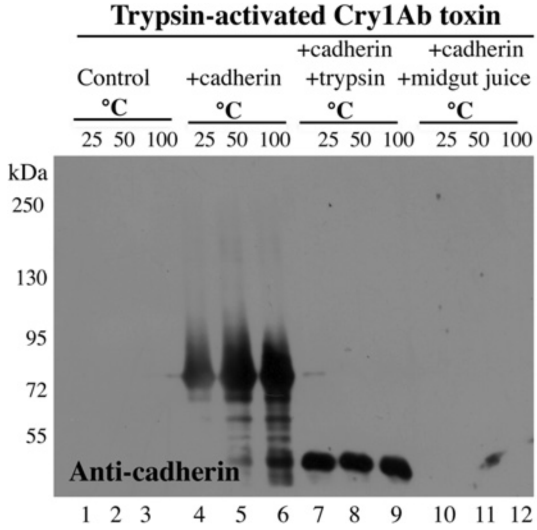

Figure S2 Detection of cadherin after incubation for 1 h with trypsin-activated monomeric Cry1Ab toxin with the cadherin fragment in the presence or absence of trypsin or midgut juice proteases from *M. sexta* at 37 °C

After incubation, samples were heated for 5 min at different temperatures and revealed in Western blot assays with anti-cadherin antibody. Lanes 1–3, control Cry1Ab toxin; lanes 4–6, incubation of Cry1Ab toxin with cadherin fragment; lanes 7–9, incubation of Cry1Ab toxin with cadherin fragment in the presence of trypsin; lanes 10–12, incubation of Cry1Ab toxin with cadherin fragment in the presence of midgut juice. Molecular masses are indicated in kDa.

[1] Correspondence may be addressed to either of these authors (email mario@ibt.unam.mx or bravo@ibt.unam.mx).

© 2014 The Author(s) The author(s) has paid for this article to be freely available under the terms of the Creative Commons Attribution Licence (CC-BY) (http://creativecommons.org/licenses/by/3.0/) which permits unrestricted use, distribution and reproduction in any medium, provided the original work is properly cited.

Biochemical Journal www.biochemj.org

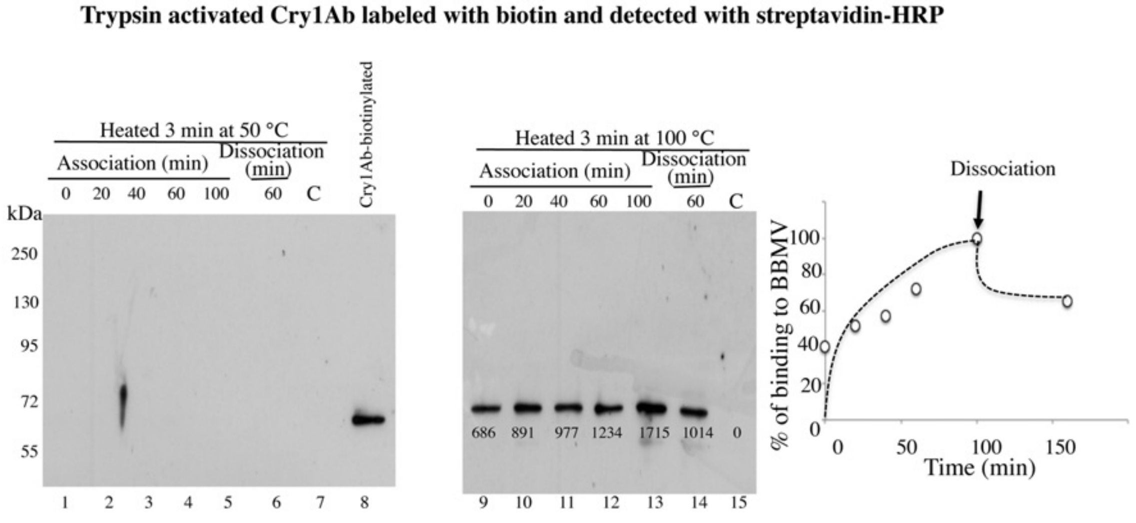

Figure S3 Association and dissociation binding assays of biotin-labelled Cry1Ab toxin bound to BBMVs from *M. sexta* larvae

Cry1Ab toxin was labelled with biotin, using biotinyl-*N*-hydroxysuccinimide ester, according to the manufacturer's instructions (GE Healthcare). A 10 μg amount of BBMV protein was incubated in binding buffer with 5 nM biotinylated Cry1Ab toxin during time-dependent association up to 100 min (lanes 1–5 and 9–13). After this time, an excess of 500-fold unlabelled toxin was added and time-dependent dissociation was analysed for up to 60 min (lanes 6 and 14). Unbound toxin was removed by centrifugation at $\sim$ 18400 ***g*** for 10 min and the membrane pellet containing the bound toxin was washed once with same buffer. The final pellet was suspended in Laemmli sample buffer and heated for 3 min at 50°C (lanes 1–8) or 100°C (lanes 9–15) before resolving by SDS/PAGE. Lanes 7 and 15 are control BBMVs without toxin incubation. Lane 8 is control biotinylated Cry1Ab toxin. Proteins were electrotransferred on to PDVF membranes, and labelled protein was detected with streptavidin coupled to horseradish peroxidase (HRP). Densitometric analysis of the bands in the blot was carried out using ImageJ software to quantify binding. These values were plotted as the percentage of binding against time. Only data from blots obtained after heating at 100°C were used in the plot, since biotinylated toxin could not be detected in the samples that were heated at 50°C which form oligomeric structures, suggesting that biotin is inaccessible to be recognized by the streptavidin protein. C, control BBMVs without toxin incubation. Molecular masses are indicated in kDa.

Received 31 October 2013/13 January 2014; accepted 23 January 2014
Published as BJ Immediate Publication 23 January 2014, doi:10.1042/BJ20131408

© 2014 The Author(s) The author(s) has paid for this article to be freely available under the terms of the Creative Commons Attribution Licence (CC-BY) (http://creativecommons.org/licenses/by/3.0/) which permits unrestricted use, distribution and reproduction in any medium, provided the original work is properly cited.
